# Trait variation along elevation gradients in a dominant woody shrub is population-specific and driven by plasticity

**DOI:** 10.1093/aobpla/plx027

**Published:** 2017-06-19

**Authors:** Alix A. Pfennigwerth, Joseph K. Bailey, Jennifer A. Schweitzer

**Affiliations:** a Department of Ecology and Evolutionary Biology, University of Tennessee, 569 Dabney Hall, Knoxville, TN 37996-0001, USA

**Keywords:** Climate change, common garden, elevation gradient, genetic variation, intraspecific variation, phenotypic plasticity, plant traits, *Rhododendron maximum*

## Abstract

Elevation gradients are frequently used as space-for-time substitutions to infer species’ trait responses to climate change. However, studies rarely investigate whether trait responses to elevation are widespread or population-specific within a species, and the relative genetic and plastic contributions to such trait responses may not be well understood. Here, we examine plant trait variation in the dominant woody shrub, *Rhododendron maximum*, along elevation gradients in three populations in the South Central Appalachian Mountains, USA, in both field and common garden environments. We ask the following: (i) do plant traits vary along elevation? (ii) do trait responses to elevation differ across populations, and if so, why? and (iii) does genetic differentiation or phenotypic plasticity drive trait variation within and among populations? We found that internode length, shoot length, leaf dry mass, and leaf area varied along elevation, but that these responses were generally unique to one population, suggesting that trait responses to environmental gradients are population-specific. A common garden experiment identified no genetic basis to variation along elevation or among populations in any trait, suggesting that plasticity drives local and regional trait variation and may play a key role in the persistence of plant species such as *R. maximum* with contemporary climate change. Overall, our findings highlight the importance of examining multiple locations in future elevation studies and indicate that, for a given plant species, the magnitude of trait responses to global climate change may vary by location.

## Teaser

Within a species, plant traits may vary substantially along environmental gradients. However, is such variation (1) consistent across locations and (2) genetic or non-genetic (i.e., plastic) in nature? In this study, we combine field observations and a common garden experiment to assess Rhododendron maximum trait variation within and among three elevation gradients. Our findings reveal that trait variation along environmental gradients in this species is (1) highly population-specific and (2) driven primarily by non-genetic factors (i.e., plasticity). Overall, our findings highlight the importance of examining multiple locations and suggest that trait responses to environmental change vary by location.

## Introduction

Contemporary climate change is altering the availability of resources and habitats critical to plant performance (Parmesan and Yohe 2003). In a rapidly changing climate, the ability of a plant species to acclimate via phenotypic plasticity or undergo genetic change will play a key role in that species’ persistence (Walther *et al.* 2002; Franks *et al.* 2014; Nicotra *et al.* 2015). Examining how a plant species’ traits respond plastically or genetically to existing climatic gradients is therefore critical for understanding and predicting whether and how plants may persist *in situ* despite a changing climate (Chevin and Lande 2010; Nicotra *et al.* 2010; Anderson and Gezon 2015).

Phenotypic plasticity and genetic change have inherently unique characteristics and limitations with respect to potential climate change response. Plasticity is recognized for its potential key role in short-term adaptive responses to rapid environmental change because it can allow a plant to both maximize fitness under optimal conditions and tolerate stressful environments in suboptimal conditions (Gianoli 2004; Hendry 2016). Plasticity may also be advantageous at local spatial scales where genetic differentiation may be hindered by gene flow (Kawecki and Ebert 2004; Hamann *et al.* 2016). However, plasticity is inherently costly (reviewed in DeWitt *et al.* 1998) and may therefore be most important as a short-term response before genetic changes occur (Gienapp *et al.* 2008). Because genetic responses are typically slow relative to plastic responses, adaptive evolution will likely be most effective with climatic change that is gradual and below a critical threshold (Lynch and Lande 1993; Aitken *et al.* 2008).

Natural climatic gradients associated with elevation are frequently used as space-for-time substitutions to infer potential trait responses to temporal climate change (Fukami and Wardle 2005; Körner 2007; Sundqvist *et al.* 2013; Read *et al.* 2014). These gradients encompass spatial variation in climatic factors including temperature, precipitation, and growing season length and should thus present strong environmental or selective pressures on plant traits that mirror contemporary climate change pressures (Dunne *et al.* 2004). Moreover, natural climatic gradients capture temporal scales (i.e. multiple plant generations) that are typically difficult to capture in experimental climate manipulations, allowing insight into both long-term and short-term responses (Grinnell 1924; Dunne *et al.* 2004; Fukami and Wardle 2005; Sundqvist *et al.* 2013).

Performance-related foliar, morphological, and phenological plant traits are highly sensitive to climatic environment (Medeiros and Ward 2013; Pratt and Mooney 2013) and may genetically or phenotypically ‘track’ climatic variation associated with elevation (Whittaker 1956; Fukami and Wardle 2005; Sundqvist *et al.* 2013; reviewed in Read *et al.* 2014). For example, plants at higher elevations have lower growth rates, smaller and thicker leaves, higher leaf nutrient content per unit area, later bud break, and earlier senescing phenology (Clausen *et al.* 1940; Oleksyn *et al.* 1998; Körner 2003; Vitasse *et al.* 2009; Bresson *et al.* 2011; Read *et al.* 2014). Common garden experiments, which identify the genetic basis of *in situ* trait variation by exposing plants sourced from different elevations (i.e. environments) to a single environment, have attributed such trait variation to genetic differentiation, plasticity, or both mechanisms (Hoffmann *et al.* 2009; Anderson and Gezon 2015; reviewed in Read *et al.* 2014).

The use of natural spatial gradients, including elevation, as climate change proxies may be confounded by fine-scale environmental heterogeneity, covarying abiotic or biotic factors, or historical site attributes of the gradient (Dunne *et al.* 2004; Primack *et al.* 2009, De Frenne *et al.* 2013; Lenoir *et al.* 2013; Kooyers *et al.* 2015). The magnitude of trait variation with climatic factors along elevation may thus vary across locations or populations, yet most studies have examined trait responses to elevation at a single location. For example, in a recent meta-analysis of leaf trait responses to elevation within and among species (Read *et al.* 2014), 75 % of studies examining intraspecific trait responses did so at only one location. Few studies have explicitly asked whether, and why, trait responses to elevation are or are not consistent across multiple populations or locations (but see Morecroft *et al.* 1992; Kooyers *et al.* 2015). Thus, to understand whether patterns of trait variation are widespread or idiosyncratic within a species and ensure accurate ecological and evolutionary inferences, it is critical to study trait variation and potential confounding environmental factors along elevation at multiple locations.

Here, we examined genetic- and plastic-based trait variation along elevation in three geographically distinct populations of *Rhododendron maximum*, a dominant, native woody shrub of eastern North America. We selected *R. maximum* because it is geographically widespread, occurs from 0 to 1800 m a.s.l., and often forms continuous populations across steep elevation gradients (Gleason and Cronquist 1991). Specifically, we addressed three questions: (i) do plant traits vary along elevation gradients? (ii) does trait variation along elevation differ among populations, and if so, why? and (iii) does genetic divergence or phenotypic plasticity drive trait variation within and among populations? To address the first question, we examined variation in eight quantitative leaf, stem, and phenology traits (internode diameter, internode length, shoot length, leaf area, leaf dry mass, specific leaf area [SLA], leaf nitrogen [N] content, leaf bud break phenology) in three *R. maximum* populations occurring along geographically distinct elevation gradients. We predicted that plant traits become increasingly ‘conservative’ at higher elevations in response to colder temperatures and shorter growing seasons (i.e. shorter and smaller internodes and shoots, smaller and lighter leaves, lower SLA and leaf N content, later bud break). We addressed the second question by exploring the relative importance of five climatic, edaphic, and topographic variables (mean annual temperature, annual precipitation, soil N, soil carbon:nitrogen [C:N] ratio, and slope) on trait variation within and among populations and predicted that the magnitude of trait responses to elevation varies across populations due to environmental and/or population genetic differentiation. To address our third question, we planted and measured traits on replicate cuttings of the individuals sampled in natural field populations in a common garden. Consistent with past theoretical and empirical work (Kawecki and Ebert 2004; Hamann *et al.* 2016), we predicted that local trait variation (i.e. along elevation within each population) is driven by plasticity, while regional variation (i.e. among populations) is driven by genetic differentiation.

## Methods

### Focal species and study sites

**Figure 1.**
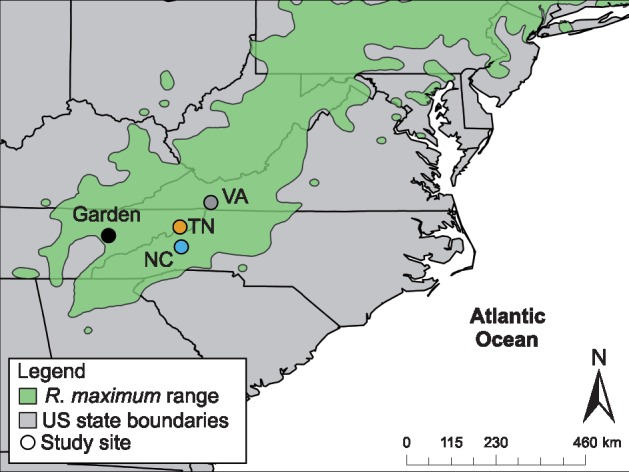
*Rhododendron maximum* elevation gradients and common garden are situated throughout the South Central Appalachian region of the *R. maximum* range (shown in green). Locations of the North Carolina (NC, blue symbol), Tennessee (TN, orange symbol), and Virginia (VA, grey symbol) elevation gradients and the University of Tennessee common garden (Garden, black symbol) are shown.

*Rhododendron maximum* (Ericaceae) is a long-lived, evergreen woody shrub widely distributed throughout eastern North America (Fig. 1; Gleason and Cronquist 1991). It is a dominant forest understory component across millions of hectares of temperate forest in South Central Appalachia, USA (Clinton 2004), the broad mountain region in which the study sites are located (Fig. 1).

We selected elevation gradients at three locations to examine *R. maximum* trait variation based on the following criteria: (i) a steep elevation gradient (>400 m total elevation change) that presents a gradient of mean annual temperature falling within the range of regional climate change projections the next ∼100 years (+1 to +5°C, scenarios RCP2.6 and RCP8.5; IPCC 2014), (ii) a continuous *R. maximum* population across the gradient, and (iii) a north- to northeast-facing aspect to minimize environmental and trait variability due to exposure. The three sites are located in: (i) George Washington and Jefferson National Forest, Virginia (36.6900°N, 81.6364°W; hereafter, ‘VA population’); (ii) Cherokee National Forest, Tennessee (36.1413°N, 82.2756°W; hereafter, ‘TN population’); and (iii) Pisgah National Forest, North Carolina (35.7230°N, 82.2461°W; hereafter, ‘NC population’) (Fig. 1). Collectively, gradients represent a total elevation change of ∼800 m. All three sites were established as National Forest within 4 years of one another (1916–1920; USFS 1973). Site descriptions are provided in Table 1.

**Table 1:** Site characteristics for each of three *Rhododendron maximum* populations sampled in this study, including the number of *R. maximum* individuals sampled (*N*_individual_), gradient aspect, the range of elevation, and the soil taxonomic classes present along each gradient. Soil taxonomic class was extracted from the USDA-NRCS Web Soil Survey database (http://websoilsurvey.nrcs.usda.gov/app/ (21 June 2017)).

Transect	*N*_individual_	Aspect	Elevation (m)	Soil taxonomic class
North Carolina	30	N	895–1584	Humic Dystrudepts, Humic Hapludults, Oxyaquic Humedepts, Typic Dystrudepts, Typic Humudepts
Tennessee	30	NE	842–1467	Humic Dystrudepts, Lithic Humudepts, Typic Dystrudepts, Typic Hapludults
Virginia	30	NE	816–1250	Fluventic Dystrudepts, Typic Hapludults

### Environmental gradient quantification

To examine potential environmental drivers of *R. maximum* trait variation within and among sites, we collected data on climatic, edaphic, and topographic variation. Using GPS coordinates obtained at each individual sampling location (Oregon 650t, Garmin, Olathe, KS, USA), we extracted (i) elevation above sea level and slope from digital elevation models in ArcMap 10.1 (Esri, Redlands, CA, USA) and (ii) interpolated mean annual temperature (MAT) and annual precipitation (AP) data, representative of 1960–1990 conditions, from the WorldClim database (Hijmans *et al.* 2005) at the highest resolution available (30 arc-seconds).

To characterize edaphic conditions in each population, we measured total soil N and soil carbon:nitrogen (C:N) ratio along each gradient. We collected and pooled three soil samples (2 ×10 cm) at each of ten elevation levels along each transect (*n *= 30 total soil samples) and quantified total soil N and C:N using dynamic flash combustion elemental analysis (Flash Elemental Analyzer 1112, Thermo Fisher Scientific, Waltham, MA, USA).

### Trait variation in natural field populations

We collected trait data in late July through early August 2014 during the *R. maximum* active growing season. Along each gradient, we sampled three *R. maximum* individuals at each of ten elevation intervals of approximately 50 m, for a total of 30 sampled individuals per site (*N* = 3 individuals × 10 elevation intervals × 3 sites = 90 total individuals). Sampled *R. maximum* individuals were separated by >40 m to minimize the probability of sampling siblings or clones.

We assessed stem morphology in the field by measuring internode and elongating shoot length and internode diameter of two outer canopy stems per individual. Internode length and diameter were averaged across the three fully developed internodes directly below the elongating shoot. To assess leaf traits, we harvested the two most recently produced but fully expanded leaves from an outer canopy leaf whorl to assess leaf area, leaf dry mass, SLA, and leaf N content. Field fresh leaves were stored at 0°C and transported to the lab, then scanned to calculate total fresh leaf area (WinFOLIA 2011a, Regent Instruments, Canada). Leaf dry mass was recorded after leaves were oven-dried at 70°C for 72 h. Specific leaf area was calculated as leaf area/leaf dry mass. Leaf area, dry mass, and SLA were averaged at the individual level. Dried leaves were ground to a fine powder (8000D Mixer/Mill, SPEX SamplePrep, Metuchen, NJ, USA) and analysed for total leaf N content with dynamic flash combustion elemental analysis (Flash Elemental Analyzer 1112, Thermo Scientific, Waltham, MA, USA).

### Genetic-based trait variation in common garden

To estimate the genetic contributions to *R. maximum* trait variation measured in the field, we established an outdoor common garden (281 m elevation, 14.4˚C MAT; 125.7 cm AP) at the University of Tennessee, Knoxville, Tennessee, USA (Fig. 1; 35.9579°N, 83.9248˚W). During field sampling in late July through early August 2014, we harvested ten terminal shoot cuttings (∼15 cm in length) from each *R. maximum* individual that we sampled (*N *= 900 cuttings). The cuttings were kept moist and transported at 0°C to a greenhouse, where we scored the lower 5 cm of each cutting using a razor, dipped the scored section in a root-inducing growth hormone (Hormodin 3, Olympic Horticultural Products, Mainland, PA, USA) and planted each cutting in potting media consisting of equal parts peat moss and perlite. We removed all but three terminal leaves and uniformly cut the remaining three leaves to 5 cm length to minimize evaporative moisture loss and encourage root growth. After a 6-month rooting period on a mist bed (misted with tap water every 15 min) we transplanted all living, rooted cuttings (*N *= 504; rooting rate = 56 %) into individual, randomized 1-gallon pots filled with the same peat-perlite mixture in an outdoor common garden. Cuttings were watered to field capacity and given biweekly fertilizer treatments (200 ppm of Peters Professional 21-7-7 Acid Special, Everris NA, Dublin, OH, USA) during the growing season to minimize non-genetic (maternal-like) effects associated with clone cuttings (Roach and Wulff 1987). To further minimize maternal-like effects, we grew cuttings in a common environment for 16 months before measuring traits on new growth (since cutting) only. By exposing individuals from diverse environments to a single environment, common gardens minimize environmentally induced plasticity and effectively expose the genetic basis of complex, quantitative trait phenotypes (Clausen *et al.* 1940; Dunne *et al.* 2004; Fukami and Wardle 2005; Anderson *et al.* 2014; De Villemereuil *et al.* 2016).

In December 2015, after 16 months of growth in common conditions, we measured average internode diameter, shoot length, and internode length and sampled one fully mature leaf per cutting to quantify leaf area, dry leaf mass, and specific leaf area on all rooted, surviving cuttings (*n = *142, 176, and 144 cuttings [mortality rate = 6 %, 5 %, 14 %] from NC, TN, and VA transects, respectively) to quantify genetic-based trait variation. We additionally measured spring leaf bud break (flushing) phenology throughout the growing season as the earliest day on which leaf bud scales opened and any emerging new leaf was visible; these data were collected every second day continually until all cuttings had flushed. Sample sizes vary among traits measured in the common garden (i.e. shoot length, but not internode length, could be measured on plants that did not produce multiple new nodes); this information is provided in Table 4.

### Statistical analysis

***Environmental gradient characterization***. To explore whether the three *R. maximum* populations experience similar environmental gradients along elevation, we built linear models predicting variation in five climatic, edaphic, and topographic variables with population (NC, TN, or VA), elevation (m a.s.l.), and the interaction between population and elevation as fixed effects. Separate models were built for MAT, AP, slope, soil N, and soil C:N. Each variable was transformed prior to analysis as needed to increase conformance to normality. We calculated ANOVA tables using partial sums of squares and assessed significance using F statistics after controlling for familywise error rates using Holm’s sequential Bonferroni procedure (e.g. α = 0.05/(*n* – significance rank + 1), where *n *=* *number of tests; Holm 1979). We calculated standardized beta coefficients for elevation based on correlations between trait and elevation z-scores (obtained by scaling and centreing untransformed data on zero).

***Trait variation in natural field populations*** **.** To quantify patterns of within-population trait variation (along elevation) and among-population variation, we used linear models. We included population (NC, TN, or VA), elevation (m a.s.l.), and the interaction between population and elevation as fixed effects. We included population as a fixed effect because we are specifically interested in the variation in trait responses to elevation explained by population. Separate models were built for each trait measured in the field, and traits were transformed prior to analysis as needed to increase conformance to normality. We calculated ANOVA tables using partial sums of squares and, using F statistics, assessed significance after controlling for familywise error rates (Holm 1979). We calculated post-hoc Tukey’s pairwise differences to compare mean trait values among populations and calculated standardized beta coefficients for elevation based on correlations between trait and elevation z-scores.

***Genetic-based trait variation in common garden*** ***.*** To quantify patterns of genetic-based trait differentiation along elevation and among populations, we used a maximum likelihood, mixed modeling approach implemented with the R package lme4 (Bates *et al.* 2015). Source elevation (m a.s.l.), source population (NC, TN, or VA), and their interaction were included as fixed effects. Because each field-sampled individual was represented by replicated cuttings in the common garden, individual identity was included as a random effect. Again, separate models were built for each trait, and traits were transformed prior to analysis as needed. Chi-square tests were performed to assess significance for each fixed effect after controlling for familywise error rates (Holm 1979). We calculated post-hoc Tukey’s pairwise differences to compare mean trait values among populations and calculated standardized beta coefficients for elevation based on correlations between trait and elevation z-scores.

***Environmental contributions to trait variation*** **.** To examine the relative importance of climatic, edaphic, and topographic factors in driving significant trait variation along elevation, we used multiple regression analysis. Specifically, we built models predicting variation in traits that exhibited significant variation along elevation in field or common garden environments in the trait analyses above, using MAT, AP, slope, soil N, and soil C:N as fixed effects. If significant population x elevation effects were detected above (and/or an overall elevation effect was driven by a single population), we conducted analyses only for those populations exhibiting significant responses to elevation. Using F statistics, significance was assessed at α = 0.05. Traits were transformed prior to analysis as needed to increase conformance to normality.

All statistical analyses were conducted in R 3.2.1 (R Foundation for Statistical Computing, Vienna, Austria).

## Results

### Environmental gradient characterization

**Figure 2.**
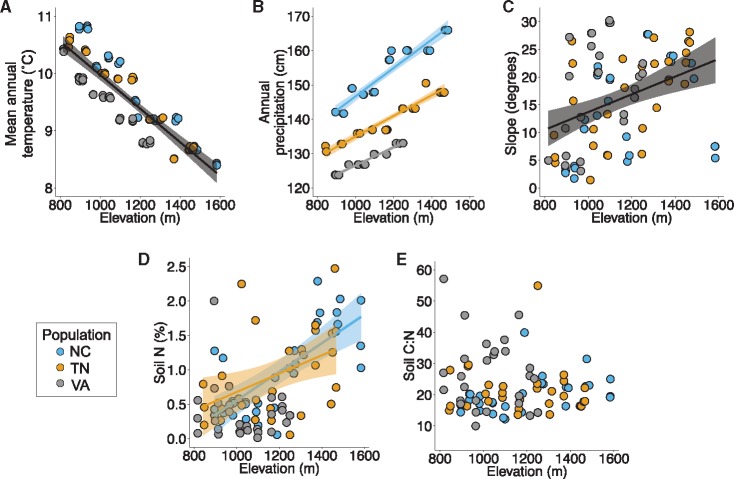
Variation in five climatic, topographic, and edaphic variables along elevation in three *Rhododendron maximum* populations (North Carolina [NC, blue]; Tennessee [TN, orange]; Virginia [VA, grey]). A solid black line indications a significant (*P *< 0.05) effect of elevation across populations (no population-by-elevation interaction). When statistical models detected significant population-by-elevation interactions, coloured regression lines corresponding only to those populations in which traits varied significantly (*P *< 0.05) along elevation are shown. The shaded region around each line represents the 95 % confidence interval for that regression.

We detected significant effects of elevation and population, but not their interaction, on MAT and slope (Table 2), indicating that mean values of these variables differ across sites but that the three gradients present similar changes in MAT and slope. On average, MAT decreases 1.9°C (Fig. 2A) and slope increases 18° (Fig. 2C) along increasing elevation across sites. We found significant main and interactive effects of population and elevation on AP and soil N (Table 2), indicating that mean values of these variables, as well as the magnitude of their change along elevation, differ by population. Due to the significant interaction, we ran separate population-specific models for each variable (using elevation as a fixed effect). We found that AP increases with elevation on average 28.0, 17.2, and 13.4 cm in the NC, TN, and VA populations, respectively (Fig. 2B). Soil N increases on average 0.76 % and 1.1 % with elevation in the NC and TN populations, respectively, and does not vary significantly with elevation in the VA population (Fig. 2D). Finally, we detected a significant effect of population, but not elevation, on soil C:N (Table 2) indicating that soil C:N differs on average across populations but does not vary significantly with elevation (Fig. 2E).

**Table 2:** Statistics for linear models incorporating elevation, transect, and their interaction as fixed effects, predicting variation in five climatic, edaphic, and topographic variables (mean annual temperature (MAT), annual precipitation (AP), topographical slope (slope), soil nitrogen (N) content, and soil carbon:nitrogen (C:N) ratio) along elevation in three natural *R. maximum* populations. Variation in these environmental variables was examined for potential importance on elevational trait variation. *F*-values (*F*) and associated degrees of freedom are displayed for each effect; standardized beta coefficients (*β*) and standard error (*SE*) are shown for elevation. Beta coefficients of elevation are not shown for those traits on which a significant population × elevation interaction effect was detected; see Results section for population-specific effects of elevation on these variables. Significant (α* *= 0.05, corrected with Holm’s sequential Bonferroni procedure) *F*-values are shown in bold; statistical significance is denoted by the following: *P < 0.05, **P < 0.01, ***P < 0.001, ****P <0.0001.

Environmental Variable	Effect				
	Population	Elevation			Population*Elevation
	*F_5,84_*	*β*	*SE*	*F_5,84_*	*F_5,84_*
MAT (˚C)	125.11****	−1.04	0.04	1329.16****	0.23
AP (cm)	1052.82****	–	–	1186.66****	5.18**
Slope (degrees)	4.65*	0.36	0.15	25.30****	0.54
Soil N content (%)	7.37**	–	–	23.03****	4.20*
Soil C:N ratio	3.57*	0.25	0.16	0.34	2.82

### Trait variation in natural field populations

**Figure 3.**
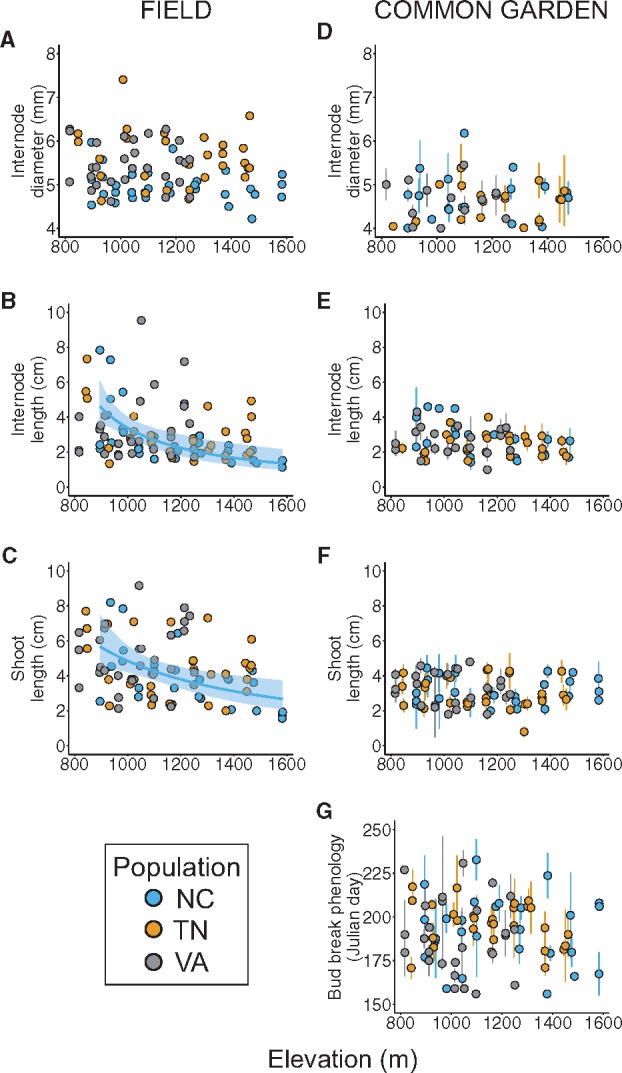
The effect of elevation on traits related to growth and timing (internode diameter, internode length, shoot length, leaf bud break phenology) in *Rhododendron maximum* measured at the individual level in natural field populations along three elevation gradients (A-C; bud break phenology was not measured in the field) and the effect of source elevation on mean ± SE trait values measured at the replicated cutting level in a common garden (D-G). Populations are represented by each of three colours (North Carolina [NC], blue; Tennessee [TN], orange; Virginia [VA], grey). A solid black line indications a significant (*P *< 0.05) effect of elevation across populations (no population-by-elevation interaction). When statistical models detected significant or marginally significant population-by-elevation interactions, coloured regression lines corresponding only to those populations in which traits varied significantly (*P *< 0.05) along elevation are shown. The shaded region around each line represents the 95 % confidence interval for that regression.

**Figure 4.**
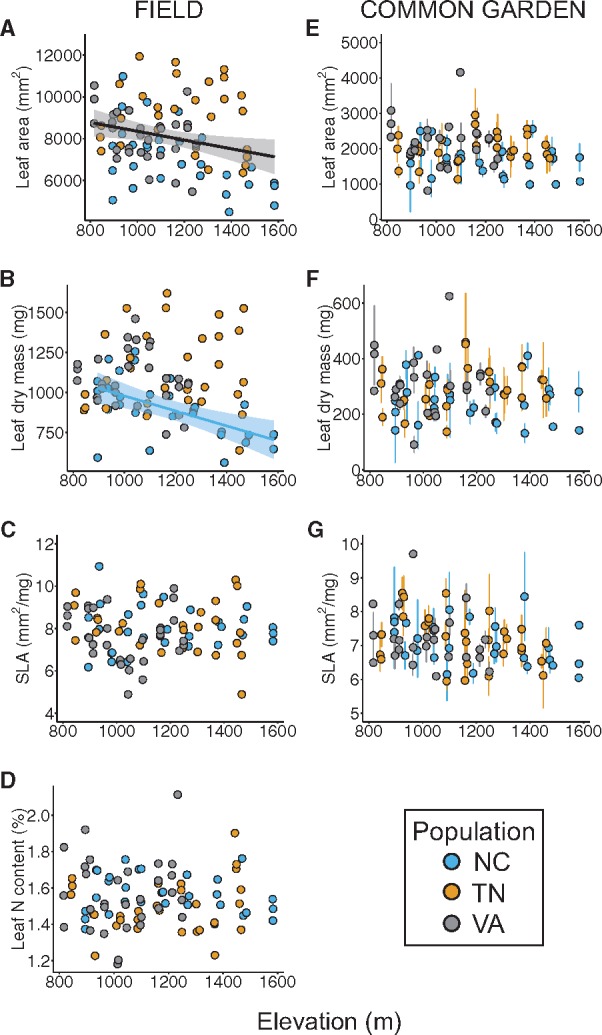
The effect of elevation on leaf-level traits (leaf area, leaf dry mass, specific leaf area [SLA], leaf nitrogen [N] content) in *Rhododendron maximum* measured at the individual level in natural field populations along three elevation gradients (A–D) and the effect of source elevation on mean ± SE trait values measured at the replicated cutting level in a common garden (E-G; leaf N content was not measured in the common garden). Populations are represented by each of three colors (North Carolina, blue; Tennessee, orange; Virginia, grey). A solid black line indications a significant (*P *< 0.05) effect of elevation across populations (no population-by-elevation interaction). When statistical models detected significant or marginally significant population-by-elevation interactions, colored regression lines corresponding only to those populations in which traits varied significantly (*P *< 0.05) along elevation are shown. The shaded region around each line represents the 95 % confidence interval for that regression.

The interaction between population and elevation was significant for internode length and shoot length (Table 3) and marginally significant for leaf dry mass (*P *= 0.053), indicating that the magnitude of response to elevation in these traits varies by population. Thus, for these traits, we ran separate models for each population (using elevation as a fixed effect) to examine how trait values varied along elevation at each location. Internode length, shoot length, and leaf dry mass significantly decreased with elevation in the NC population only (*R*^2^= 0.56, 0.54, 0.33; *F*_1,28_ = 14.94, 19.77, 13.94; *P *< 0.0001, 0.0001, 0.001; respectively), with internodes on average 3-fold shorter, shoots 5-fold shorter, and leaves 20 % lighter (i.e. lower mass) at highest elevations relative to lowest elevations (Figs 3B, C and4B). We detected a significant main effect of elevation, but no elevation-by-population effect, for leaf area (Table 3), with leaves becoming smaller with increasing elevation (Fig. 4A). However, elevation explained relatively little variation in this trait (*R*^2^* *= 0.06), and we found that leaf area decreased significantly in the NC population only (*R*^2^* *= 0.22, *F*_1,28_* *= 9.36, *P *= 0.005), with leaves being on average 14 % smaller at highest elevations relative to lowest elevations. We identified no main or interactive effect of elevation on internode diameter, SLA, or leaf N content (Figs 3A, 4C and D).

**Table 3:** Statistics for linear models, incorporating population, elevation, and their interaction as fixed effects, of seven functional trait values along elevation at three field transect locations containing sampled *Rhododendron maximum* populations (*N *= 90 sampled *R. maximum* individuals). *F*-values (*F*) and associated degrees of freedom (*DF*) are displayed for each effect; standardized beta coefficients (*β*) and standard error (*SE*) are shown for elevation. Beta coefficients of elevation are not shown for those traits on which a significant population × elevation interaction effect was detected; see Results section for population-specific effects of elevation for these traits. Significant (α* *= 0.05, corrected with Holm’s sequential Bonferroni procedure) *F*-values are shown in bold; level of statistical significance is denoted by the following: *P < 0.05, **P < 0.01, ***P < 0.001.

Field trait	Effect				
	Population	Elevation			Population*Elevation
	*F_2,84_*	*β*	*SE*	*F_1,84_*	*F_2,84_*
Internode diameter (mm)	10.21***	−0.23	0.15	1.39	0.26
Internode length (cm)	2.21	–	–	17.11***	6.59**
Shoot length (cm)	0.02	–	–	16.49***	7.09**
Leaf area (mm^2^)	16.07***	−0.41	0.14	9.79**	1.15
Leaf dry mass (mg)	12.51***	−0.42	0.14	3.63	3.05
Specific leaf area (mm^2^/mg)	2.11	0.02	0.17	0.96	0.57
Leaf nitrogen content (%)	2.34	−0.09	0.17	0.13	0.50

**Table 4:** Statistics for linear mixed effects models, incorporating population of origin (population), elevation of origin (elevation), and their interaction as fixed effects, and individual identity (i.e. sampled individual in field population) as a random effect, of seven functional trait values measured on 16-month old *Rhododendron maximum* cuttings in a common garden. Sample size (*N*, number of cuttings measured) for each trait is shown. Chi-square (*X^2^*) values and associated degrees of freedom are shown for each effect; standardized beta coefficients (*β*) and standard error (*SE*) are shown for elevation.

Common garden trait	*N*	Effect				
		Population	Elevation			Population*Elevation
		$X_{\mathrm{DF = 2}}^{2}$	*β*	*SE*	$X_{\mathrm{DF = 1}}^{2}$	$X_{\mathrm{DF = 2}}^{2}$
Internode diameter (mm)	153	5.30	0.01	0.14	2.03	1.32
Internode length (cm)	150	1.93	−0.38	0.14	1.84	4.38
Shoot length (cm)	323	1.08	−0.14	0.13	0.48	0.52
Leaf area (mm^2^)	317	8.53	−0.10	0.13	0.10	3.20
Leaf dry mass (mg)	317	6.81	−0.04	0.13	0.18	2.22
Specific leaf area (mm^2^/mg)	314	0.28	−0.15	0.12	5.78	0.03
Bud break (Julian day)	315	1.588	−0.07	0.13	0.830	0.486

**Figure 5.**
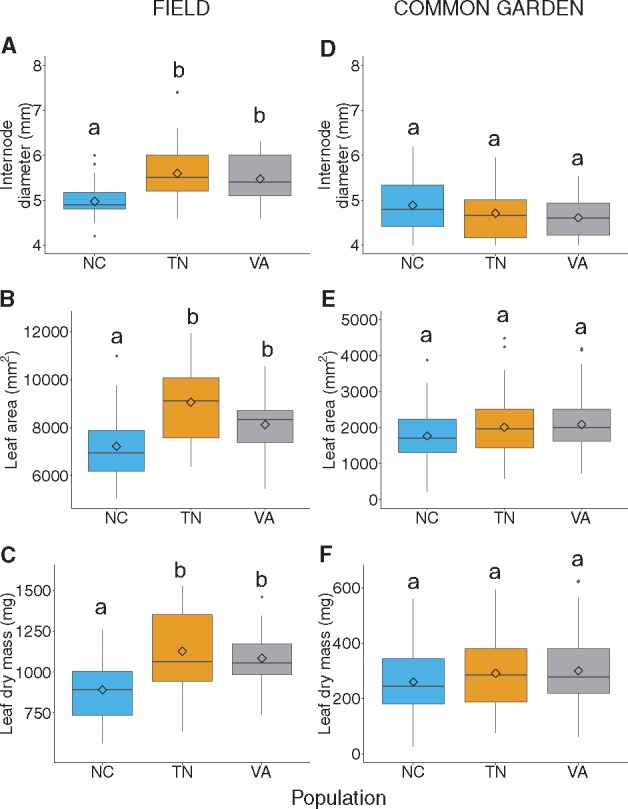
Population means of three traits (internode diameter, leaf area, and leaf dry mass) that differ across *Rhododendron maximum* populations (North Carolina, NC, blue; Tennessee, TN, orange; Virginia, VA, grey) in field (A–C) and common garden (D–F) environments. Diamond symbols and bold horizontal lines within each box represent the mean and median of the data, respectively; boxes extend to the upper and lower quartile; whiskers extend from each box to the minimum and maximum values of the data, excluding outliers; outliers are shown by black points above or below whiskers. Within each panel, boxes that do not share the same letter are significantly different from each other (Tukey test *P *< 0.05).

We found a significant effect of population on internode diameter, leaf area, and leaf dry mass (Table 3), indicating population-level differentiation in mean phenotypic values of these traits. Individuals in the NC population had, on average, 12 % and 8 % smaller internode diameters, 22 % and 13 % less leaf area, and 22 % and 17 % lighter leaves than plants in the TN and VA populations, respectively (Fig. 5A–C). We detected no main or interactive effect of population on SLA or leaf N content (Table 3).

### Genetic-based trait variation in common garden

Source elevation had no significant main or interactive effect on common garden values for any trait, indicating no genetic basis to clinal trait variation in these traits (Table 4; Figs 3 and 4). Source population also had no significant main or interactive effect on any trait in the common garden (Table 4), indicating absence of genetic differentiation among populations for these traits.

### Environmental contributions to trait variation

Multiple regression analysis indicated that variation in MAT alone explains significant variation in internode length (*R*^2^* *= 0.42, *F*_1,25_ = 10.68; *P *= 0.003) and shoot length (*R*^2^* * =  0.45, *F*_1,25_ = 10.71; *P *= 0.003) in the NC field population, whereas variation in soil N explains significant variation in leaf dry mass in this field population (*R*^2^* *= 0.24, *F*_1,25_ = 4.34; *P *= 0.05). Although we detected a significant response of leaf area to elevation in the field that appears to be driven by the NC population, the environmental variables we analysed do not explain significant variation in this trait overall or within the NC population individually (*P *> 0.05, all variables). Similarly, no environmental variable explained significant variation in common garden SLA values (*P *> 0.05, all variables).

## Discussion

Elevation gradients are commonly used to examine intraspecific trait variation in the context of climate change. However, studies have rarely examined whether environmental gradients and/or trait responses to these gradients are consistent across locations. Moreover, the relative genetic and plastic contributions to trait clines along elevation are unknown for many species. Through field observations of the dominant woody shrub *R. maximum* across three elevation gradients, we showed that traits vary along elevation in only one of the three gradients. A paired common garden experiment indicated that plasticity underlies trait variation within and among populations. Here, we discuss that these findings suggest (i) trait responses to environmental variation in one population may not sufficiently reflect a species’ universal response and (ii) plasticity may be an important mechanism underlying *R. maximum* persistence through climatic change.

### Trait variation along elevation is population-specific

In partial support of our expectations, four of seven traits exhibited ‘conservative’ responses to increasing elevation (Cordell *et al.* 1998; Pérez-Harguindeguy *et al.* 2013) in natural *R. maximum* populations. However, these trait responses were generally restricted to the NC population, and three of seven traits exhibited no significant response to elevation, suggesting that the effect of environmental gradients along elevation can be variable and population-specific. This may be because the climatic, edaphic, and topographic gradients we examined appear to co-vary the most with elevation in the NC population (see Fig. 2). Additionally, the gradient in annual precipitation was about 63 % and 109 % steeper in the NC population than the VA and TN populations, respectively. Plants in this population may therefore be experiencing a relatively consistent environmental signal along elevation and/or a stronger moisture gradient, leading to stronger clinal trait responses. Edaphic gradients, in particular, appear to be less consistent in the VA and TN populations, which may cause a countergradient effect (Conover and Present 1990; Conover and Schultz 1995) and lead to ‘noise’ in clinal trait variation along climatic gradients associated with elevation at these locations.

Fine-grained spatial variation in such factors as topography, surface hydrology, canopy cover, or wind effects, can be particularly important for understory plant species (Geiger *et al.* 2003; Graae *et al.* 2012; De Frenne *et al.* 2013; Lenoir *et al.* 2013; Opedal *et al.* 2015) and could explain why traits did not vary along elevation in the VA and TN populations. Additionally, variation in biotic interactions with herbivores, pathogens, or mutualists can strongly influence trait phenotypes (Gross *et al.* 2009; Bardgett and Wardle 2010). Thus, *R. maximum* traits in this study may be responding to a mosaic of fine-scale abiotic and biotic variation in the understory, rather than to a single, linear gradient along elevation. These findings highlight the potential complexity associated with elevation gradients, and suggest that the utility of these gradients as spatial proxies for temporal climate change may be oversimplified. However, such fine-grained environmental heterogeneity may ultimately prove beneficial for understory plant species such as *R. maximum* in the context of climate change, creating micro-refugia where species might persist locally despite increasingly unfavorable conditions overall (Lenoir *et al.* 2013). Future work examining trait variation with respect to microclimatic conditions specific to the understory (i.e. understory light level, soil moisture, and temperature) as well as biotic interactions could provide further insight into the ecological complexity confounding the use of elevation gradients as climate change proxies and elucidate the extent of potential climate change buffering for understory species like *R. maximum.*

We have shown that climatic and/or edaphic gradients can differ even across sites that occur in the same region, have similar land-use histories, and contain similar plant communities, and that these differences may lead to population-specific trait responses to elevation. However, most studies examining intraspecific plant trait responses to elevation, including 75 % of those reviewed by Read *et al.* (2014), have not done so across multiple sites. Among studies that examined multiple populations, population-specific effects of elevation on at least some traits appear common (i.e. Morecroft *et al.* 1992; Byars *et al.* 2007; Premoli and Brewer 2007; Hoffmann *et al.* 2009; Gonzalo-Turpin and Hazard 2009; Fajardo and Piper 2011; Kooyers *et al.* 2015). For example, Kooyers and co-authors (2015) found that the directional effect of elevation on four of ten traits in *Mimulus guttatus* was reversed with increasing latitude due to variation in growing season length, temperature, and seasonal water availability across sites. The general lack of replication in this field, coupled with evidence from our study and others for population-specific trait responses, therefore underscores the need for ongoing examination of the consistency, or lack thereof, of trait responses to elevation among populations.

### Plasticity drives trait variation within and among populations

Trait variation along elevation within natural populations, as well as trait variation among populations, did not persist in the common garden, indicating a general lack of genetic basis to the trait variation examined. These findings corroborate previous work demonstrating extensive plasticity in foliar and growth-related traits of *R. maximum* (Nilsen 1986; Nilsen and Bao 1988) and other *Rhododendron* species (Hébert *et al.* 2011; Niinemets *et al.* 2003) to light, moisture, or temperature manipulations. The lack of genetic differentiation within populations along elevation may be due to high gene flow (Kawecki and Ebert 2004; but see Kameyama *et al.* 2001 and Hirao *et al.* 2006 for examples of restricted gene flow in *Rhododendron*). *Rhododendron maximum* seeds are wind-mediated or passively dispersed (Hille Ris Lambers *et al.* 2005), and pollination is accomplished primarily by bees (Romancier 1971). Thus, assuming no phenological barriers to reproduction, these two processes may generate sufficient gene flow within populations to effectively hinder adaptive genetic divergence from occurring along environmental gradients associated with elevation (Savolainen *et al.* 2007; Kremer *et al.* 2014).

Phenotypic plasticity can be adaptive in fine-grain spatially or temporally heterogeneous environments by buffering performance and enabling plants to persist through large environmental shifts and/or to colonize novel environments (Moran 1992; Ernande and Dieckmann 2004; Lind and Johansson 2007; Chevin and Lande 2010; Baythavong 2011; Hendry 2016). Given that *Rhododendron maximum* populations in the southern Appalachian region have both persisted and increased in frequency and growth following several regional forest disturbances, including heaving logging, *Castanea dentata* (American chestnut) blight (Boring *et al.* 1981; Plocher and Carvell 1987; Dobbs and Parker 2004, Elliott and Vose 2012), and, more recently, non-native insect invasion causing widespread mortality of *Tsuga canadensis* (eastern hemlock; Ford *et al.* 2012), it is possible that *R. maximum* populations in this region have evolved increased plasticity in response to past environmental heterogeneity and frequent, severe disturbance events. Regardless of its evolutionary source, our findings of phenotypic plasticity along elevation gradients presenting temperature gradients representative of regional climate change projections, suggest that plasticity will likely continue to play an important role for *R. maximum* persistence with ongoing temporal climate change.

In the current study, we did not detect a strong genetic basis to within- or among-population variation in several traits; however, future work involving additional experimental approaches at different spatial scales would further address the importance of genetic variation in this species. First, sampling populations along a latitudinal gradient across the species’ range could increase our power to detect genetic variation across populations at larger spatial scales. Reciprocal transplants both within sites (from high to low elevation and *vice versa*) and among populations would expose plants to the natural field conditions each population has experienced and could elucidate the relative importance of local adaptation and/or adaptive plasticity at varying spatial scales in this species (Kawecki and Ebert 2004; Blanquart *et al.* 2013). Finally, molecular analyses to characterize the genetic makeup of these populations could provide further insight into the various evolutionary processes (i.e. gene flow, drift, selection, or plasticity) underlying trait variation in this system.

## Conclusions

Our findings suggest that multiple populations within a species may not exhibit a single, universal response to climatic variation, highlighting the importance of sampling multiple populations to avoid over- or underestimating possible trait responses. Further, our results indicate phenotypic plasticity will likely play an important role in allowing *R. maximum* to persist locally despite a changing climate. Ongoing work to identify the relative roles of climatic, edaphic, and biotic environments driving understory plant trait variation could provide insight into why trait patterns do or do not exist along environmental gradients and elucidate the extent to which plants will respond to, or be buffered from, global climate change. Finally, we suggest that a deeper level of sophistication in elevation studies, including replicated observations across multiple populations, will allow us to continue drawing robust climate change inferences from these natural spatial gradients.

## Sources of Funding

The National Science Foundation Graduate Research Fellowship Program under Grant No. DGE-1452154, the Department of Ecology and Evolutionary Biology at the University of Tennessee, Knoxville, and a Research Grant from the American Rhododendron Society supported this research.

## Contributions by the Authors

A.A.P. performed the research, collected and analysed all data, and was the primary writer of the manuscript. All authors contributed to the study design, interpreted analysis results, and contributed substantially to revisions.

## Conflict of Interest Statement

None declared.
